# Oslo HEMS Conference 2023

**DOI:** 10.1186/s13049-023-01135-4

**Published:** 2023-12-04

## I1 Abstracts from the Oslo HEMS Conference 2023 Scientific Sessions

### Sole Lindvåg Lie^1^, Hans Morten Lossius^1^, Marius Rehn^1^

#### ^1^Norwegian Air Ambulance Foundation, Oslo, Norway

##### **Correspondence:** Sole Lindvåg Lie (sole.lindvaag.lie@norskluftambulanse.no)

*Scand J Trauma Resusc Emerg Med* 2023, **31(Suppl 2)**:I1

In a historic first, the European HEMS community gathered for the Oslo HEMS Conference (O-HEMS-C) December 4 to 6, 2023, in Oslo, Norway. This event serves as a nexus for professionals dedicated to prehospital care, drawing clinicians, researchers, and industry representatives from across the Globe.

Scientific abstracts lie at the heart of O-HEMS-C, playing a pivotal role in advancing our understanding of prehospital critical care. Abstracts serve as a foundation for knowledge to achieve best clinical practice in our field. The abstract committee is proud to present the best abstracts in this Supplement of Scandinavian Journal of Trauma, Resuscitation and Emergency Medicine. They provide a rich tapestry of insights. Thematically they spanned from experimental studies in both humans and animals to investigations in trauma system, novel rescue techniques, systematic literature reviews and clinical trials.

As an event that happens every two years, alternating with the London Trauma Conference, we look forward to welcoming an even greater number of contributors and abstracts at the next gathering in Oslo on December 1 to 3, 2025.

## A1 How should tranexamic acid be administered in haemorrhagic shock? – Continuous serum concentration measurements in a swine model

### Trine Lynghaug^1^, Håkon K Bakke^2^, Ole M Fuskevåg^3^, Eirik W Nielsen^4^, Erik S Dietrichs^5^

#### ^1^UiT, The Arctic University of Norway, Anaesthesia and Critical Care Research Group, Department of Clinical Medicine, Faculty of Health Sciences, IKM, Tromsø, Norway; ^2^University Hospital of North Norway, Department of anaesthesia and critical care, Tromsø, Norway; ^3^UiT, The arctic University of Norway, Department of Clinical Medicine, Faculty of Health Sciences, Tromsø, Norway; ^4^University Nord, Bodø, Norway; ^5^University of Oslo, Department of Oral Biology, Oslo, Norway

##### **Correspondence:** Trine Lynghaug (trine.lynghaug@outlook.com)

*Scand J Trauma Resusc Emerg Med* 2023, **31(Suppl 2)**:A1

**Background** Tranexamic acid (TXA) reduces mortality in trauma patients. Intramuscular (i.m.) administration could be advantageous in low-resource and military settings. Achieving the same serum concentration as i.v. administration is important to achieve equal mortality reduction. Therefore, we aimed to investigate whether dividing an i.m. dose of TXA between two injection sites, and whether an increase in dose, would lead to serum concentrations comparable to those achieved by i.v. administration.

**Methods** Norwegian landrace pigs (n = 29) from a course in haemostatic emergency surgery were given tranexamic acid 1 h after start of surgery. Blood samples were drawn at 0, 5,10, 15, 20, 25, 35, 45, 60 and 85 min. The samples were centrifuged and serum TXA concentrations quantified with liquid chromatography–tandem mass spectrometry (LC–MS/MS). The use of two injection sites was compared to distributing the dose on one injection site, and a dose of 15 mg/kg was compared to a dose of 30 mg/kg. All i.m. groups were compared to i.v. administration. The research animals were registered in the Norwegian Food Safety Authority´s audit and application system, FOTS, and their use approved by the Norwegian Food Safety Authority.

**Figure 1 (abstract A1):**
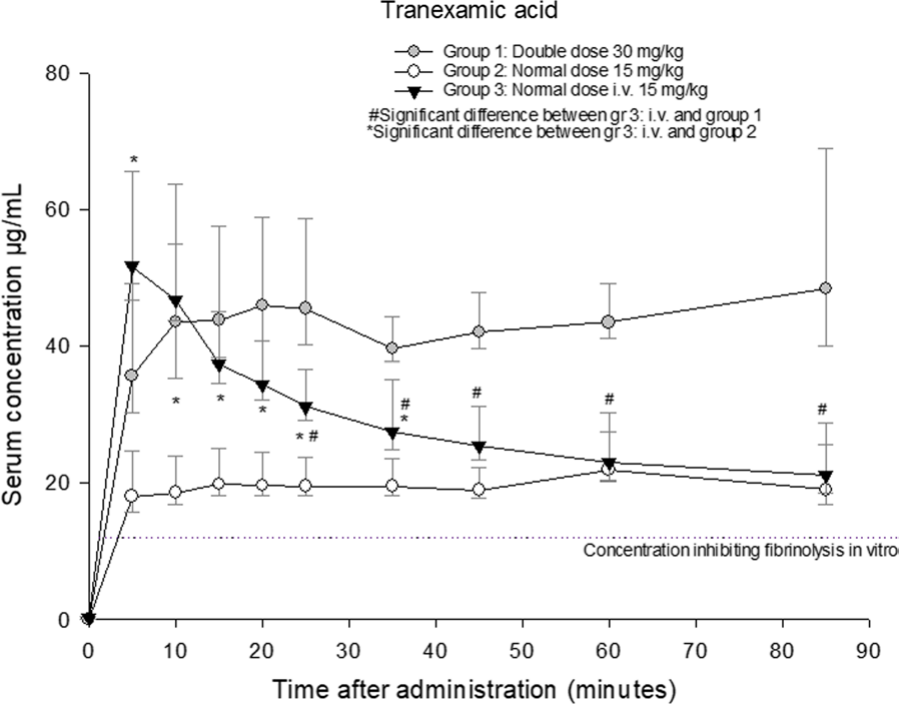
Development of TXA concentration in the three groups with double and normal dose intramuscular versus normal dose intravenously.

**Results** The groups were in a similar degree of shock. Increasing the i.m. dose from the standard of 15 mg/kg to 30 mg/kg resulted in significantly higher serum concentrations of TXA, comparable to those achieved by i.v. administration. Distributing the i.m. dose on two injection sites did not affect drug-uptake, as shown by equal serum concentrations. Figure 1 shows the development of the TXA serum concentrations throughout the experimental protocol.

**Conclusion** For i.m. administration of TXA, 30 mg/kg should be the standard dose. With a short delay, i.m. administration will provide equal serum concentrations as i.v. administration, above what is considered necessary to inhibit fibrinolysis.

## A2 The effect of a vapor barrier in combination with active external rewarming for patients with accidental hypothermia

### S. Mydske^1^, G. Brattebø^2^, Ø. Østerås^2^, Ø. Wiggen^3^, J. Assmus^1^, Ø. Thomassen^1^

#### ^1^Mountain Medicine Research Cluster, The Norwegian Air Ambulance Foundation, Norway; ^2^Department of anesthesia and intensive care, Haukeland University Hospital, Bergen, Norway; ^3^SINTEF Digital, Health Research, Trondheim, Norway

##### **Correspondence:** S. Mydske (sigurd.mydske@norskluftambulanse.no)

*Scand J Trauma Resusc Emerg Med* 2023, **31(Suppl 2)**:A2

**Background** Use of a vapor barrier in prehospital care of cold-stressed or hypothermic patients aims to reduce evaporative heat loss and accelerate rewarming rate. Application of vapor barriers is recommended in various guidelines along with both insulating and wind/waterproof layers and a source of active external rewarming, but evidence of its effect is limited. Our aim was to investigate the effect of using a vapor barrier as the inner layer in the recommended “burrito” model for wrapping hypothermic patients in the field.

**Methods** 16 healthy volunteers wearing wet clothing were subjected to a 30 min cooling period in a snow chamber before being wrapped in a model including an active heating source either with a vapor barrier (intervention) or without (control) following a randomized crossover design. Mean skin temperature, core temperature and humidity in the model were measured, and the humidity sensor was placed outside the vapor barrier in the intervention group to assess how effective the barrier was at containing vapor. Shivering intensity and thermal comfort was assessed using a subjective questionnaire. Mean skin temperature was our primary outcome, and humidity and thermal comfort were secondary outcomes. Primary outcome data was analyzed using analysis of covariance (ANCOVA).

**Results** Our results indicate a significant increase in mean skin temperature in the vapor barrier group (intervention) compared to the group without a vapor barrier (control). We could see higher levels of humidity and temperature in the control group compared to outside the vapor barrier in the intervention group, meaning the vapor barrier successfully contained the vapor and latent heat closer to the patient limiting evaporative heat loss. There also seems to be a trend of higher thermal comfort in the intervention group compared to the control group.

**Conclusion** The use of a vapor barrier in combination with an active external heat source leads to higher mean skin rewarming rates in patients wearing wet clothing at risk of accidental hypothermia.

*Trial registration* ClinicalTrials.gov identifier: NCT05779722.

## A3 Tube Tip In Pharynx (TTIP) ventilation – a simple rescue technique in limited resource settings: A Case Report

### Sandra Ellefsen^1^, Anja B. Stubager^2^, Michael S. Kristensen^2^

#### ^1^Department of Anesthesia and Intensive Care Medicine, St. Olavs University Hospital; ^2^Department of Anesthesia and Operating Theatre Services, Copenhagen University Hospital, Rigshospitalet, Copenhagen, Denmark

##### **Correspondence:** Sandra Ellefsen (sandra.ellefsen@stolav.no)

*Scand J Trauma Resusc Emerg Med* 2023, **31(Suppl 2)**:A3

**Figure 2 (abstract A3):**
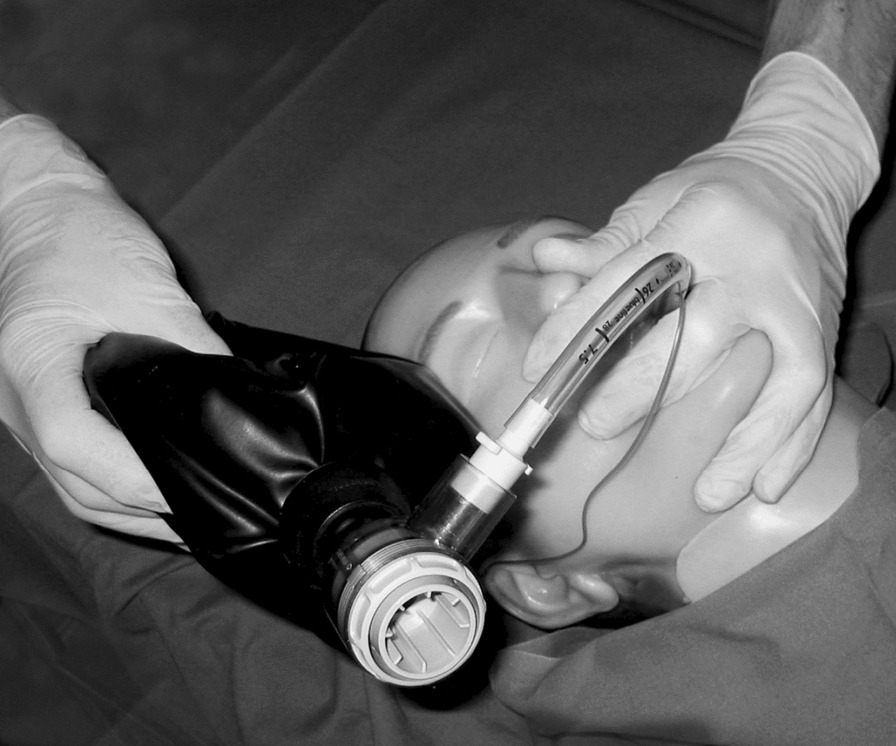
Tube Tip In Pharynx technique.

**Background** Airway control is a cornerstone in anesthesiology. However, airway devices may be scarce in limited resource situations, such as prehospital settings or austere environments. Tube Tip In Pharynx (TTIP) ventilation is a simple one-handed airway rescue technique intended to obtain airway patency if bag-valve-mask (BVM) ventilation or endotracheal intubation fails. The technique requires a standard cuffed endotracheal tube and a self-inflating bag and is performed by placing the tube via the mouth or nose with the tip in the pharynx (Figure 2), inflating the cuff to push the base of the tongue away from the surrounding tissue and using one hand to lift the chin and enclose the patient’s mouth and nose to create a seal.

**Methods** Case report reported according to the CARE guidelines. Informed consent from the patient is obtained.

**Results** The case was experienced by a Danish nurse anesthetist working for Médecins Sans Frontières in Nigeria. The patient was a male in his mid-forties with an estimated body-mass-index of 40 and unknown medical history. He had suffered a gunshot to the abdomen and needed an urgent laparotomy. Preoxygenation was performed with a self-inflating resuscitation bag with 5 L/min of 100% oxygen. General anesthesia was induced with thiopental and suxamethonium. Available airway equipment included Macintosh blade 3 and 4, a rigid stylet and endotracheal tubes in 6.0 mm and 7.0 mm size. The first intubation attempt failed. BVM also failed with subsequent rapid desaturation. The nurse anesthetist then performed the TTIP technique successfully and normal oxygen saturation was restored. Three additional attempts to intubate via direct laryngoscopy failed, and the TTIP technique was used to maintain oxygenation between the attempts. Airway management was solved by a surgical airway, performed while the patient was ventilated with the TTIP technique.

**Conclusion** TTIP is a simple, one-handed technique which may contribute to airway control in a life-threatening situation. It is easy to learn, takes seconds to perform and requires only readily available equipment. Hence, it may be a useful skill in the prehospital setting, where airway equipment is limited.

## A4 Protocol deviation frequency as a means of measuring study implementation quality

### Randi Simensen^1^, Liv-Jorunn Smalberget^2^, Fridtjof Heyerdahl^3^

#### ^1^University of Oslo, Oslo, Norway; ^2^Division of Prehospital Services, Innlandet Health Trust, Moelv, Norway; ^3^Air Ambulance Department, Oslo University Hospital, Oslo, Norway

##### **Correspondence:** Randi Simensen (randi.simensen@norskluftambulanse.no)

*Scand J Trauma Resusc Emerg Med* 2023, **31(Suppl 2)**:A4

**Background** Randomized control trials (RCT) are complex, and the ethical considerations and urgency of patient care add to these challenges in the prehospital setting. Few, if any, of Emergency medical service (EMS) study workers in the PreMeFen study had any prior experience in collecting research data but were thoroughly trained. We aimed to assess changes in protocol deviation (PD) frequency as a measurement of study implementation quality and compare these changes to the EMS providers' motivations.

**Methods** We collected information on all protocol deviations recorded as part of the PreMeFen study. We also conducted a survey among the trained EMS study workers, where we asked about their motivation prior to, during and after the study period with a 10 points Likert scale. All answers were recorded anonymously.

**Figure 3 (abstract A4):**
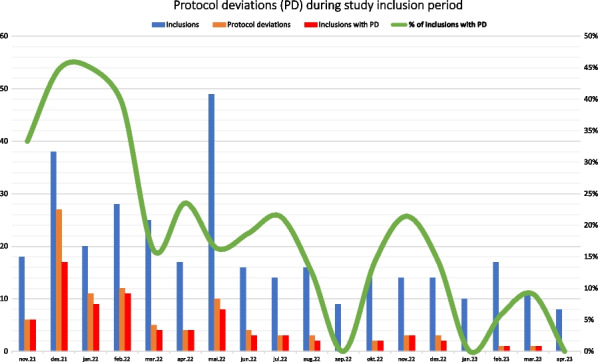
Inclusions with protocol deviations as percentage of number of inclusions.

**Results** From November 2021 to May 2023, in total 77 EMS providers included 338 patients. The mean inclusion rate was 27.8 per month in the first seven months, decreasing to 11.5 per month in the last 10 months. The total number of PDs were 95, and 76 (22%) of the inclusions had one or more deviations. The percentage of inclusions with PD was on average 41% the first four months, declining to 4% the last four months of the study (Figure 3). 67 (87%) of 77 study workers completed the survey. The motivation at the launch of the study was median (25–75 Percentile) 8 (8–10). During the study, the motivation fell to 7 (5–8), and the end-of-study motivation to participate in a next study increased to 8 (7–9).

**Conclusion** The implementation quality seemed to increase over the study period with a PD reduction from 41 to 4% during the study. Although we measured a very high motivation at the launch of the study, the high PD rate confirms the complexity of the study with a corresponding drop in motivation. Inclusion volume, guidance from the study management and repeated training decreased the PD rate, increased the quality with a corresponding motivation increase at the end of study.

*Trial registration* The study was registered in ClinicalTrials.gov (ref. NCT05137184) 30 November 2021, and EudraCT (ref. 2021–000549–42).

## A5 Trauma patient transport using HEMS or EMS in Sweden – a comparison of mortality and prehospital time

### Oscar Lapidus^1^, Rebecka Rubenson Wahlin^1^, Denise Bäckström^2^

#### ^1^Department of Clinical Science and Education, Södersjukhuset, Karolinska Institutet, Sweden; ^2^Department of Biomedical and Clinical Sciences, Linköping University, Sweden

##### **Correspondence:** Oscar Lapidus (oscar.lapidus@ki.se)

*Scand J Trauma Resusc Emerg Med* 2023, **31(Suppl 2)**:A5

**Background** The benefits of helicopter emergency medical services (HEMS) transport of adults following major trauma have been examined with mixed results, with some studies reporting a survival benefit compared to regular emergency medical services (EMS). As the benefit of HEMS in the context of the Swedish trauma system remains unclear, this study aimed to investigate differences in mortality and prehospital time intervals for trauma patients in Sweden transported by HEMS compared to road ambulance EMS.

**Methods** A total of 74 032 trauma patients treated during 2012–2022 were identified through the Swedish Trauma Registry (SweTrau). The primary outcome was 30-day mortality; secondary outcomes were Glasgow Outcome Score (GOS) at discharge from hospital (to home or rehab), proportion of patients with ISS ≥ 16 who triggered a trauma team activation (TTA) on arrival to hospital and the proportion of patients with ISS ≥ 16 and GCS ≤ 8 who were subject to endotracheal intubation.

**Figure 4 (abstract A5):**
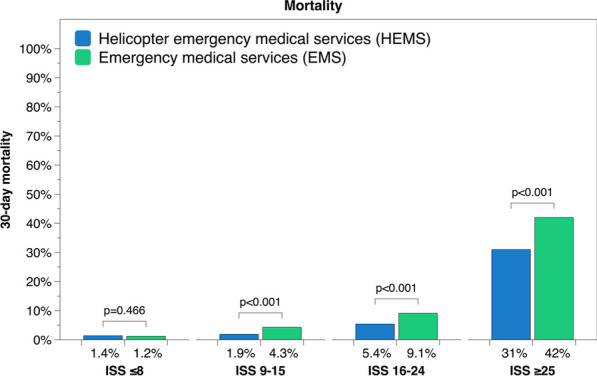
30-day mortality for patients transported by HEMS and EMS.

**Results** 4 529 out of 74 032 patients were transported by HEMS during the study period. HEMS patients had significantly lower mortality compared to patients transported by EMS at 1.9% vs 4.3% (ISS 9–15), 5.4% vs 9.4% (ISS 16–24) and 31% vs 42% (ISS ≥ 25) (p < 0.001) (Figure 4). Transport by HEMS was also significantly associated with worse neurological outcome at discharge from hospital (p < 0.001), as well as a higher rate of in-hospital trauma team activation (TTA) for patients with ISS ≥ 16 and higher rate of prehospital intubation for patients with ISS ≥ 16 and a prehospital GCS ≤ 8. Total prehospital time was longer for patients transported by HEMS compared to EMS at 63 vs 53 min (p < 0.001).

**Conclusion** Trauma patients transported to hospital by HEMS have significantly lower mortality compared to those transported by EMS, despite longer prehospital time intervals and higher injury severity. However, the survival benefit may be at the expense of a higher degree of adverse neurological outcome. Transport using HEMS may be the preferrable option for transport of severely injured trauma patients in Sweden, but further investigations of HEMS vs EMS in different prehospital systems are desirable.

## A6 Induction of Pre-Hospital Emergency Anaesthesia i-PHEA: a national survey of UK HEMS practice

### Mark Hodkinson^1^, Kurtis Poole^1^

#### ^1^Thames Valley Air Ambulance, Stokenchuch, Oxford, UK

##### **Correspondence:** Mark Hodkinson (mark.hodkinson@tvairambulance.org.uk)

*Scand J Trauma Resusc Emerg Med* 2023, **31(Suppl 2)**:A6

**Background** Pre-hospital emergency anaesthesia is a critical intervention undertaken by helicopter emergency medical teams. Previous studies informed current practice for induction regimes, using a standardized approach of fentanyl, ketamine and rocuronium. There may be a trend towards post-induction hypotension attributed to the induction regime used. Several new combinations of fentanyl, ketamine and rocuronium are emerging in clinical practice. There is currently no consensus on what induction regimes should be used.

**Methods** A semi-structured survey was distributed to the medical leads of all UK air ambulance organisations between December 2022 and February 2023. The survey sought to establish provision of pre-hospital emergency anaesthesia and current induction regimes for stable, unstable and post-cardiac arrest patients. Data was extracted from Microsoft Forms into Excel and reported using descriptive statistics. The survey was endorsed by the National HEMS Research and Audit Forum.

**Results** 19 air ambulance organisations responded (response rate 86%). Most organisations provide over 100 pre-hospital emergency anaesthetics per annum (79%, n = 15/19). The survey confirmed that a combination of fentanyl, ketamine and rocuronium is used for induction of anaesthesia, with an increasing trend towards practitioner choice as opposed to prescribed drug regimes. In haemodynamically unstable patients, the overwhelming regime used is that of ketamine and rocuronium (53%, n = 10/19). There is variability in the dose of rocuronium from 1 mg/kg to 2 mg/kg. There was no consensus between the respondents for the induction regimes used in patients with return of spontaneous circulation.

**Conclusion** There remains variability in the approach to pre-hospital emergency anaesthesia. There is a growing dataset that would enable development of a registry to better understand induction regimes and the impact on patient physiology. Organisations are increasingly adopting a patient centered, practitioner choice model towards induction of anaesthesia. This demonstrates maturity and experience of pre-hospital services. Further work on the favourable dose of rocuronium is warranted.

## A7 A fatal methaemoglobinemia after deliberate ingestion of sodium nitrite

### Marit Bekkevold^1^, Sten Frøyshov^2^, Morten Rostrup^2^, Fridtjof Heyerdahl^1^

#### ^1^Air Ambulance Department, Oslo University Hospital, Oslo, Norway; ^2^Department of Acute Medicine, Oslo University Hospital, Oslo, Norway

##### **Correspondence:** Marit Bekkevold (marit.bekkevold@norskluftambulanse.no)

*Scand J Trauma Resusc Emerg Med* 2023, **31(Suppl 2)**:A7

**Background** Severe methaemoglobinemia after self-poisoning is rare, but with a media focus on “suicide kits” containing sodium nitrites sold on internet, prehospital health care personnel should be aware of the condition.

**Methods** We present a case with a suicidal ingested of sodium nitrite. The patient’s relatives have consented to this publication.

**Results** A 28-Year-old woman called the medical emergency dispatch centre after having ingested 25 g sodium nitrite purchased from an online service. She had slurred speech, felt nauseous, and the call was then lost. Paramedics arrived after 12 min and found her unconscious with cyanosis from head to mid-thorax and insufficient ventilation. Helicopter emergency medical crew (HEMS) arrived 23 min after the call. Pulse was 120/minute and oxygen saturation was 75% despite 12 L/min oxygen and mask-bag ventilation. She was uneventfully intubated using ketamine and rocuronium, and evacuation was initiated. She had bradycardia 25–27/min 14 min after intubation. She was helicopter-transported with ongoing cardiopulmonary resuscitation (CPR) and received methylene blue on the helipad immediately after landing at the hospital. At arrival, she had pulseless electric activity changing to ventricular fibrillation. Methaemoglobin was high (Table 1) and the blood was chocolate brown. The hospital treatment additionally included transfusions and vasopressors without return of spontaneous circulation. Four hours after intake she was established on VA-ECMO and spontaneous circulation was later obtained. However, head CT scan revealed global oedema and herniation, and she died the next day.

**Conclusions** Sodium nitrite is a food preservative also sold as suicide kit on several internet platforms. It oxidizes haemoglobin (Hb) to methaemoglobin (MetHb) which cannot transport oxygen, leading to tissue hypoxia. Dark brown blood (chocolate cyanosis) can be used diagnostically if a blood gas analysis is not available. The antidote methylene blue is important to reduce MetHb back to Hb. In our case, the HEMS physician consulted clinical toxicologist on the way to the patient, which eased the decision to minimize the time on site, although the patient needed CPR en-route, and the HEMS physician could communicate the need of methylene blue brought to the helipad before arrival at the hospital.

**Table 1 (abstract A7):** Blood gas analyses.

Time	21:19	22:04	22:44	00:27	03:23	08:08
pH	7.16	7.05	6.95	7.26	7.56	7.56
pCO_2_	2.0	3.0	2.9	4.0	2.9	3.5
pO_2_		9.8	70	66.7	59.9	26.8
BE	-22	-24	-28	-14	-3	1
HCO_3_^−^	5.1	6.2	4.7	13.5	19.5	23.3
Lactate	28	27	30	26	13.6	7.4
Methaemoglobin	high	high	0.45	0.05	0.02	0.02

## A8 Advanced haemodynamic monitoring in prehospital adult trauma patients: A systematic review

### Marie H. Dæhlen^1^, Sole L. Lie^2^, Jonny Hisdal^1^, Lars-Øivind Høiseth^3^, Marius Rehn^1^

#### ^1^Faculty of Medicine, University of Oslo, Oslo, Norway; ^2^Department of Research and Development, Norwegian Air Ambulance Foundation, Oslo, Norway; ^3^Department of Anaesthesia and Intensive Care, Division of Emergencies and Critical Care, Oslo University Hospital, Oslo, Norway

##### **Correspondence:** Marie H. Dæhlen (marie.holtet.daehlen@gmail.com)

*Scand J Trauma Resusc Emerg Med* 2023, **31(Suppl 2)**:A8

**Background** Haemorrhage is the leading cause of preventable deaths in trauma patients, and half of these cases occur during the prehospital period. Currently, precise methods to detect haemorrhage are lacking, and standard vital signs are insufficient to identify and monitor shock. As far as we know, there is no published review on advanced hemodynamic monitoring and its clinical impact in trauma patients. We therefore systemically reviewed the literature for availability and efficacy of advanced hemodynamic monitoring in prehospital adult trauma patients.

**Methods** We defined advanced hemodynamic monitoring as any monitoring device or technique that measures or estimates systemic, regional, or local blood flow, pressure, perfusion, or oxygenation, excluding standard vital signs. We searched MEDLINE, Embase, and CINAHL on October 20^th^, 2022. The search was limited to 1990 and forward, and comprised three terms: 1) Prehospital setting, 2) Advanced hemodynamic monitoring, 3) Trauma patients (≥18 years). The main outcomes were mortality, morbidity, and prehospital management. Articles were screened and identified by two independent authors in Covidence. The study follows PRISMA guidelines.

**Figure 5 (abstract A8):**
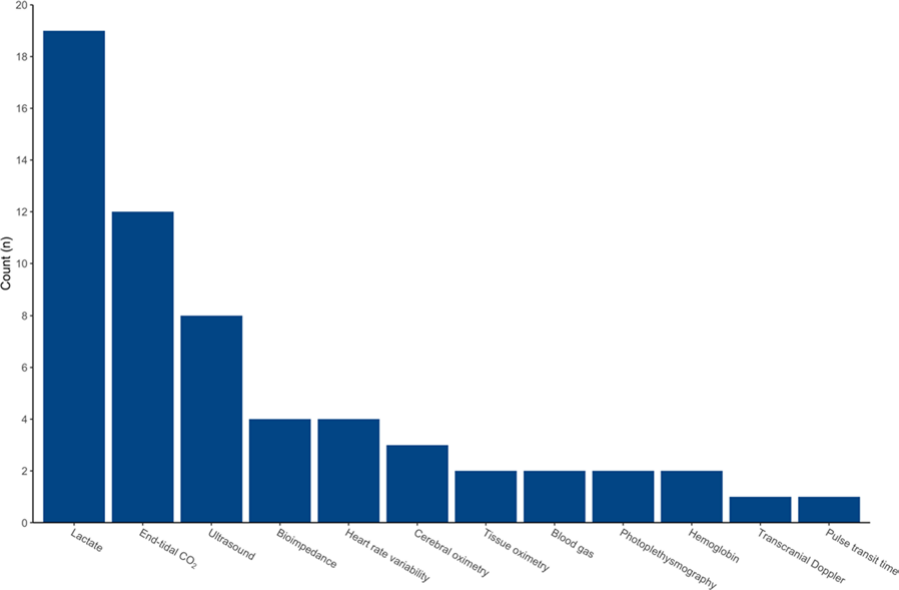
Frequency of various advanced haemodynamic monitoring techniques used in studies in prehospital adult trauma patients.

**Results** We screened 3681 and included 60 articles. Lactate (n = 19), end-tidal CO_2_ (n = 12), ultrasound (n = 8) and tissue oximetry (n = 7) were the most common advanced hemodynamic monitoring techniques (Figure 5).

**Conclusion** Various advanced haemodynamic monitoring techniques have been studied in adult prehospital trauma patients. Whether they improve clinical outcome is unclear.

*Trial registration* Registered in PROSPERO (CRD42022373051) on November 15th, 2022.

## A9 Utilising 360 degree video to deliver immersive training for the prehospital environment

### Lily Stanley^1^, Jamie Anderson^2^, Darren Best^1^

#### ^1^Thames Valley Air Ambulance, Stokenchurch, UK; ^2^Flight Beyond Sight

##### **Correspondence:** Lily Stanley (lily.stanley@tvairambulance.org.uk)

*Scand J Trauma Resusc Emerg Med* 2023, **31(Suppl 2)**:A9

**Background** Practitioners within the emergency setting have a requirement to train for high acuity low occurrence procedures and clinical scenarios. Training includes both the motor skills to perform a procedure but also the mental modeling of steps involved. Creating high fidelity simulations regularly for all staff is resource heavy, and teams may operate from multiple standby points during a shift, limiting access to training equipment. To further understand the potential benefits and limitations of high fidelity immersive training videos as a training method, a pilot 360° training video was created and delivered via virtual reality headset.

**Methods** High fidelity simulations were filmed using a 360° camera of scenarios with an initial pilot of an unanticipated difficult airway in a head injured cyclist. HEMS doctors and paramedics operated in their usual roles, whilst volunteers were used as actors to replicate the busy and noisy environment that teams encounter in reality. The footage was edited to create an immersive training video that can be viewed on a virtual reality headset based at standby points for crew use on shift.

**Results** A mixed group of 14 doctors and paramedics working at Thames Valley Air Ambulance watched the training video and gave feedback via an anonymous survey. The training video achieved a high level of realism and immersiveness, with participants giving an average rating of 8.5 out of 10. 93% of participants felt that this type of training platform could be beneficial and 86% said access to such content would make them more likely to do training when at standby points.

**Conclusion** The pilot training video was well received by participants and highlighted key benefits over conventional simulation, including the quick set-up time and on-demand nature of the content lending itself well to emergency services work. Rather than replacing simulation based training methods, we see it as a complementary resource that can be used where training time and access to equipment is limited or where knowledge refreshers are needed. We aim to build a library of immersive 360° training videos with integrated confirmation of learning that will benefit staff and ultimately their patients.

## A10 Functional outcome after trauma: a Norwegian national population-based study

### Inger Marie W Nilsbakken^1^, Torben Wisborg^2^, Stephen Sollid^3^, Elisabeth Jeppesen^4^

#### ^1^Department of Research, Norwegian Air Ambulance Foundation, Oslo, Norway; ^2^Norwegian National Advisory Unit on Trauma, Oslo University Hospital, Oslo, Norway; ^3^Prehospital Division, Oslo University Hospital, Oslo, Norway; ^4^Faculty of Health Studies, VID Specialized University, Oslo, Norway

##### **Correspondence:** Inger Marie W Nilsbakken (inger.nilsbakken@norskluftambulanse.no)

*Scand J Trauma Resusc Emerg Med* 2023, **31(Suppl 2)**:A10

**Background** There is a lack of knowledge regarding the functional outcome of patients after trauma, likely related to the high focus on mortality as outcome-measure. Norway has a diverse population distribution, where 20% of the population resides in remote areas. Previous trauma literature has shown that the nature of trauma differs between remote and urban areas. Thus, the aim of this study was to assess functional outcome after trauma in the Norwegian trauma population with an emphasis on geographical location and outcome.

**Methods** We performed a registry-based study on 34,611 patients from the Norwegian trauma registry from 2015–2020. Functional outcome was measured by discharge Glasgow Outcome Scale (GOS) and Statistics Norway’s Centralization Index was used for urban-remote classification. We used descriptive statistical methods to assess differences in study population characteristics and functional outcome, and a multinomial logistic regression model, adjusted for confounding factors, to assess the relationship between geographical location and functional outcome.

**Results** Ninety-four per cent of trauma patients had no disability or moderate disability at discharge. Eighty-one per cent of patients with severe disability or vegetative state had a New Injury Severity Score (NISS) > 15. We found that patients in remote areas as opposed to urban areas had higher odds of severe disability and vegetative state (OR 1.25, p-value = 0.05).

**Conclusion** The majority of trauma patients admitted to a trauma hospital in Norway were discharged with minimal change in functional outcome. Patients with severe injuries (NISS > 15) had the greatest decline in functional outcome. Incurring injuries in remote areas were linked to an increased likelihood of severe disability or vegetative state. This study underscores the possible significance of geographical location in influencing the likelihood of functional impairment following trauma.

## A11 Evaluation of the offset static rope evacuation procedure. Insights from a safe job analysis

### Eirik Bjorheim Abrahamsen^1^, Håvard Mattingsdal^2^, Håkon Bjorheim Abrahamsen^3^

#### ^1^University of Stavanger, Department of Safety, Economics and Planning, Stavanger, Norway; ^2^Rescue Technical Department, Norwegian Air Ambulance Helicopter, Oslo, Norway; ^3^Department of Anaesthesiology and Intensive Care, Stavanger University Hospital, Stavanger, Norway

##### **Correspondence:** Håvard Mattingsdal (haavard.mattingsdal@norskluftambulanse.no)

*Scand J Trauma Resusc Emerg Med* 2023, **31(Suppl 2)**:A11

**Background** Recently, the Norwegian Helicopter Emergency Medical Service (HEMS) has developed a procedure for a special type of static rope rescue operation, referred to as the offset technique. In this technique, the helicopter is offset from the accident site, and the HEMS rescuer uses an offset throw line to gain access to the scene. Today, there is little practical experience of such operations, and a need has been identified for more knowledge on the potential hazards encountered during this type of operation. Such knowledge is of importance for further development of the procedure for the offset technique. The objective is to identify potential hazards for helicopter rescue operations using the static rope offset technique and, thereby, to improve the procedure for such operations. This may lead to improved safety for patients and crew members during offset rescue operations.

**Methods** A Safe Job Analysis is used to identify the hazards of offset rescue operations. Such operations are divided into tasks and sub-tasks. For each sub-task, we identify potential hazards and suggest ways of preventing these.

**Results** Through the Safe Job Analysis, we suggest some changes in the existing procedure for the offset technique, to make it more robust against potential hazards.

**Conclusion** We have demonstrated the value of Safe Job Analysis for improving the static rope offset evacuation procedure. Our analysis has led to some changes in the procedure for offset rescue operations.

## A12 Characteristics of the most severely ill and injured patients in a Norwegian Helicopter Emergency Medical Service

### Eirik Ringen^1^, Helge Haugland ^2^, Jostein Rødseth Brede^2^

#### ^1^Faculty of Medicine and Health Sciences, Norwegian University of Science and Technology, Trondheim, Norway; ^2^Department of Emergency Medicine and Pre-hospital Services, St. Olavs University Hospital, Trondheim, Norway

##### **Correspondence:** Eirik Ringen (eirik.pedersen@ntnu.no)

*Scand J Trauma Resusc Emerg Med* 2023, **31(Suppl 2)**:A12

**Background** Physician-manned helicopter emergency medical services (HEMS) are dispatched to a variety of incidents, ranging from less serious to life-threatening. The skillset of a physician may be most important to utilize for the most critically ill and severely injured patients. A better understanding of these patients may be important to optimize dispatch criteria, training, and equipment setups. The aim of this study was to describe the characteristics of patients with the national advisory committee on aeronautics (NACA) score 5 and 6, primarily by diagnostic group and interventions performed.

**Methods** Retrospective cohort study on aggregated data from the HEMS-base in Trondheim, Norway. All patients with NACA score 5 and 6 in the ten-year period from 2013 to 2022 were included. Patients with return of spontaneous circulation (ROSC) after successful cardiopulmonary resuscitation were separated from non-cardiac arrest patients.

**Figure 6 (abstract A12):**
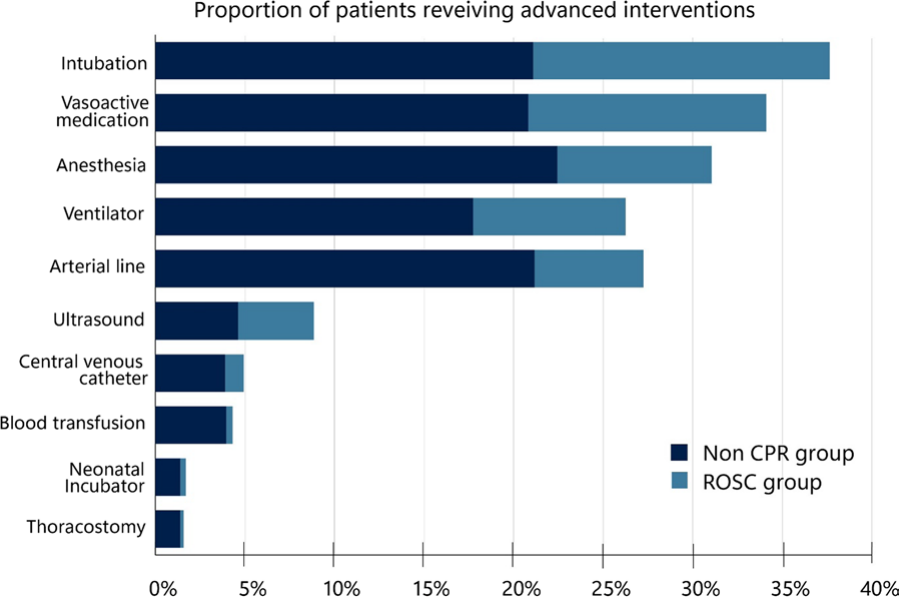
Proportion of patients receiving advanced interventions. CPR indicates cardiopulmonary resuscitation; ROSC, return of spontaneous circulation.

**Results** Out of 9546 patient encounters, 2598 patients were included, with 1640 in the NACA 5 and 958 in NACA 6 group. Patient age was median 63 (interquartile range 45–74) and 64% of the patients were male. Post-ROSC patients accounted for 24% of patients. Of the non-cardiac arrest patients, the most frequent etiology was trauma (16%), cardiac (15%), neurologic (14%) and respiratory (11%). The most common physician-requiring advanced interventions were general anesthesia (22%), intubation (21%), invasive blood pressure monitoring (21%) and ventilator treatment (18%) (Figure 6). The mean number of total interventions per mission were unchanged during the study period (5,92, SD 0,36).

**Conclusion** Twenty-seven percent of HEMS dispatches were to NACA 5 and 6 patients. Twenty-four percent of these were post-ROSC patients. Sixty-three percent of all patients received at least one advanced physician-requiring intervention.

## A13 Peripheral venous pressure waveform during simulated haemorrhage in healthy volunteers

### Aura Koistinaho^1^, Sole Lindvåg Lie^2^, Harald Lenz^3^, Leiv Arne Rosseland^1^, Jonny Hisdal^1^, Lars Øivind Høiseth^3^

#### ^1^Institute of Clinical Medicine, University of Oslo, Oslo, Norway; ^2^Department of Research and Development, Norwegian Air Ambulance Foundation, Oslo, Norway; ^3^Division of Emergencies and Critical Care, Oslo University Hospital, Oslo, Norway

##### **Correspondence:** Aura Koistinaho (a.e.koistinaho@studmed.uio.no)

*Scand J Trauma Resusc Emerg Med* 2023, **31(Suppl 2)**:A13

**Background** Early diagnosis of haemorrhage remains challenging. A recent study reported promising findings using peripheral venous pressure (PVP) waveform in anaesthetised rats, but whether this extends to awake humans remains unclear. Here, we aimed to study how simulated haemorrhage affects the peripheral venous pressure waveform amplitudes in healthy volunteers.

**Methods** Data from fifteen healthy participants enrolled in a previous study (NCT04641949) were reanalysed. All had undertaken simulated haemorrhage in 10 mmHg increments of lower body negative pressure (LBNP) every two minutes from 0 to 80 mmHg, or until haemodynamic decompensation. PVP was measured continuously in an antecubital superficial vein. The PVP was analysed using short-time Fourier transform. The amplitudes of the cardiac-synchronous oscillations were extracted and averaged for each LBNP level. Changes in PVP waveform amplitudes during LBNP were analysed using linear mixed regression.

**Results** The residuals in the regression model were right-skewed, and the amplitude results were therefore log(e)-transformed. Log(e)-amplitude (mmHg) at baseline was -2.6 (95% confidence interval (CI): -2.8 to -2.4) with a change per LBNP level of -0.11 (95% CI: -0.14 to -0.09; P < 0.001).

**Conclusion** We found statistically significant reductions in PVP waveform amplitudes during simulated haemorrhage. Although statistically significant, the clinical significance of the findings remains to be elucidated.

## A14 Ambulance and Helicopter Response Times in Emergency Medical Services. The AHRTEMIS project

### Peter Martin Hansen^1^, Marius Rehn^2^, Annmarie Touborg Lassen^3^, Anders Perner^4^, Søren Mikkelsen^5^

#### ^1^Danish Air Ambulance, Aarhus, Denmark; ^2^Norwegian Air Ambulance Foundation; ^3^Odense University Hospital, Odense, Denmark; ^4^Rigshospitalet, Copenhagen University Hospital, Copenhagen, Denmark; ^5^Prehospital Research Unit, Region of South Denmark, Odense, Denmark

##### **Correspondence:** Peter Martin Hansen (peter.martin.hansen@rsyd.dk)

*Scand J Trauma Resusc Emerg Med* 2023, **31(Suppl 2)**:A14

**Background** The purpose of the AHRTEMIS project is to enhance current knowledge on response times in criteria-based dispatch and the association with mortality, outcome and severity illness. Response time defined as the time from dispatch to patient contact for emergency medical services vehicles such as ambulances and helicopters is a key performance indicator. Often, response time is referred to in political debate on emergency medical services coverage, ambulance resource shortage and creating public security. However, moving fast and being non-compliant with traffic regulation is dangerous for patients and personnel and may be detrimental to public safety. Apart from specific conditions such as out-of-hospital cardiac arrest and trauma, there is lacking scientific evidence to justify that the shortest possible response time is positively associated with mortality and patient outcome.Therefore, the intention to describe several factors influencing time to an event such as mortality is paramount to the project. We hypothesize that overtriage is prominent and that time as a quality indicator is disputable.

**Methods** The AHRTEMIS project comprises four parts:

- A protocol article describing the AHRTEMIS project.
- A systematic literature review and meta-analysis on the association between response times and outcome measures.
- An epidemiological study on criteria-based dispatch association with interventions; on-scene time and transport time’s association with mortality.
- An epidemiological study on selected specific chapters in the criteria-based dispatch on response time’s association with mortality.

**Results** We will publish developing results from the AHRTEMIS project in peer-reviewed journals; present results at relevant international scientific conferences; we will present project progress at www.ahrtemis.dk. The accompanying Ph.D. dissertation defense is aimed for 2026.

**Conclusion** The AHRTEMIS project seeks to enhance knowledge and understanding in criteria-based dispatch and provide decision-makers with the option of performing differentiated response urgency for optimal resource utilization.

*Trial registration* We registered the systematic literature review protocol on PROSPERO on 10 September 2023 (acknowledgement of receipt [462339]). We have acquired all authorizations from relevant authorities. I.e., from Region of Southern Denmark, Secretariat and Legal Office, Journal # 23/30574 to access patient journals and from CEO secretariat, Odense University Hospital, to manage sensitive patient information, Journal # 23/31007.

## A15 Patterns in Danish major incident radio communication: Time for patching?

### Peter Martin Hansen^1^, Marius Rehn^2^, Søren Mikkelsen^3^

#### ^1^Danish Air Ambulance, Aarhus, Denmark; ^2^Norwegian Air Ambulance Foundation, Oslo, Norway; ^3^Prehospital Research Unit, Region of Southern Denmark Odense, Denmark

##### **Correspondence:** Peter Martin Hansen (peter.martyin.hansen@rsyd.dk)

*Scand J Trauma Resusc Emerg Med* 2023, **31(Suppl 2)**:A15

**Background** Radio communication in the medical management of major incidents (MI) may be compromised by technical shortcomings or human factors due to e.g., lacking introduction to communication equipment or psychological phenomena such as the Startle effect. Insufficient communication may be detrimental to command and control and has been shown to influence patient survival and outcome.

**Methods** We obtained the TETRA radio communication logs from two serious Danish MI. We investigated the radio shifts to the issued temporary talk groups by the ambulances, rapid response units and helicopters dispatched to the incidents. We evaluated if the shifts were timely and to the relevant talk group. Authorization to access communication logs was provided by responsible authorities.

**Results** The emergency medical dispatch centres dispatched 19 and 48 emergency medical services to the incidents, respectively. In the Great Belt train accident on 2 January 2019, 37.8% of the 43 radio shifts were incorrect. In the Field’s mass shooting on 3 July 2022, 34.7% of the 213 shifts were not according to the planned grid. In both MI, communication shortcomings did not influence patient outcome due to optimal incident site organization.

**Conclusions** Communication shortcomings in two Danish MI show a pattern of difficulty in radio shifts for various reasons. We propose the use of forced steering or *patching* of TETRA radios in MI encompassing e.g., more than ten units to ensure sufficient command and control.

*Trial registration* Not applicable.

